# Table-Turning and Spirit-Rapping

**Published:** 1853-10-01

**Authors:** 


### TABLE-TURNING AND SPIRIT-RAPPING.

THE aberrations of the human mind, when darkened by superstition, are almost inconceivable; it seems to fall into an abyss—a deep within the lowest deep—which is utterly unfathomable! We have before us a *brochure*, with shame be it spoken, from the pen of an English clergyman of the Church of England, who we are assured is no fictitious personage, but a *bonâ fide* preacher of the Word of God in one of the churches at Bath. If it had been handed to us printed in black letter with an old wooden binding, we should have held it up as an example of the profligate credulity and ignorance of the middle ages; or were we not assured by those who personally know this unworthy member of the church that he is in earnest, we should imagine the pamphlet, written like Dean Swift's Sermon on a Broomstick as a quiz, a satire upon one of the prevailing follies and delusions of the age. But we can find no such apology to plead in extenuation of the offence which this reverend divine has committed, which we regard with mingled feelings of pity and indignation. A table-turning mania has literally "turned his head;" hence he thus introduces himself to the notice of the public:—

"Every one has heard," it is the Rev. E. Gillson, M.A., Curate of Lyncombe and Widcombe, Bath, who now, gentle reader, addresses you! "and most people have witnessed something of table-*turning;* but, perhaps, few are prepared for the astonishing phenomena of table-*talking*. Mr. Godfrey has done a good service in his investigation of the subject, and in the account which he has published of it.* All that I shall at present attempt, is chiefly to corroborate his testimony and reiterate his warning.

* In two little works, entitled, "Table-Moving Tested, and proved to be the result of Satanic Agency;" and "Table-Turning, the Devil's Modern Masterpiece." By the Rev. N. S. Godfrey, S.C.L., of St. Catherine's Hall, Cambridge, and Incumbent of Wortley, Leeds. London: Seeley.

"I found that some members of my congregation had tried the experiment of putting questions to the table. On their first attempt, they were not prepared to expect an answer; but, to their great consternation, when a question was proposed, the table deliberately lifted up its foot and replied. Further questions were put, and an instant reply invariably given. I heard of this, and felt desirous of witnessing the phenomena, for the purpose of investigation. I therefore proposed a meeting with these friends and another family who had been accustomed to table-turning as a mere amusement. We accordingly met last Friday evening (Sept. 2, 1853), seven in number. I had never before witnessed any experiment in table-turning, and therefore requested those who had been accustomed to it to commence operations. Their hands had not been on the table many minutes before a cracking was heard, and this was immediately followed by a slight movement of a very peculiar character. *It was a sort of heaving, straining motion in the table. A question was then put, and an answer immediately given.* I placed my hand upon the table, and put a variety of questions, all of which were instantly and correctly answered. Various ages were asked, and all correctly told. In reply to trifling questions, possessing no particular interest, the table answered by quietly lifting up the leg, and rapping. But, in answer to questions of a more exciting character, it would become violently agitated, and sometimes to such a degree that I can only describe the motion by the word *frantic!*"

After the fibres of the table have been thus "heaving" and "straining" with a sort of colicky pain, and its *motion* has become *frantic*, the reverend gentleman propounds to it the following insane queries:—

"I inquired—Are you a departed spirit?

"The answer was, Yes, indicated by a rap.

"Are you unhappy?

"The table answered by a sort of *writhing* motion, which no natural power over it could *imitate.*

"It was then asked, shall you be for ever unhappy?

"The same kind of writhing motion was returned.

"*Are you a fallen angel?*

"No answer, which indicated a negative.

"*Do you know the fallen angels?—Yes.*

"*Are they more powerful than you?—Yes.*

"*Are you obliged to obey them?—Yes.*

"*Do you like their society?—Yes.*

"*Do you know Satan?—Yes.*

"*Is he the Prince of Devils?—Yes.*

"*Will he be bound?—Yes.*

"*Will he be cast into the abyss?—Yes.*

"*Will you be cast in with him?—Yes.*

"How long will it be before he is cast out?—He rapped ten.

"Will wars and commotions intervene?

"*The table rocked and reeled backwards and forwards for a length of time, as if it intended a pantomimic acting of the prophet's prediction:—'The earth shall reel to and fro like a drunkard, and shall be removed like a cottage; and the transgression thereof shall be heavy upon it; and it shall fall, and not rise again.*'"*

This is pretty well, but worse follows; the philosophical curate wishes to ascertain the "head quarters" of Satan: like another Faustus, he thus interrogates the magic table:—

"I then asked, where are Satan's head-quarters? Are they in England? There was a slight movement.

"Are they in France?—A violent movement.

* Is. xxiv. 20.

"Are they in Spain?—Similar agitation.

"*Are they at Rome?—The table literally seemed frantic.*"

The sectarian *animus* of the reverend gentleman here sufficiently unveils itself! Alas! for Rome!

"Roma—Roma! non e piu come e era prima."

"At the close of these experiments," continues the reverend performer, "which occupied about two hours, the invisible agent, in answer to some questions about himself, did not agree with what had been said before. I therefore asked,—

"Are you the same spirit that was in the table when we begun?—No.

"How many spirits have been in the table this evening?—Four.

"This spirit informed us that he had been an infidel, and that he embraced Popery about five years before his death. Amongst other questions, he was asked,—

"*Do you know the Pope?—The table was violently agitated.*

"I asked,—How long will Popery continue?

"He rapped ten; exactly coinciding with the other spirit's account of the binding of Satan."

The reverend expositor of these blasphemies subscribed, we may presume, to the Thirty-nine Articles before receiving Holy Orders; yet gives himself up to these unhallowed speculations with a freedom from which the most unblushing Atheist (if such a being exist) would recoil! Nay, he has the effrontery to introduce the Bible itself into these mountebank performances, which we are informed were actually exhibited in the Bath Theatre, and assures us that the movements of the table were stopped the moment the Bible was placed upon it.

"*The table was engaged in rapping out a number, but the instant the divine volume was laid upon it, the movement ceased. When the Bible was removed, it went on. This was repeatedly tried, and invariably with the same result. Other books were laid upon the table, similar in size and shape to the Bible, but without any effect.*"

Blessed be the memory of Ferdinand Mendes Pinto!

"Can such things be,
And overcome us like a summer cloud
Without our special wonder?"

But to proceed. The crazed curate next soliloquizes upon these prodigies in the following sublime strain:—

"What is the meaning of these things? What was my motive in investigating? and what is my object in relating them? I entered upon the inquiry, not for the gratification of mere idle curiosity, but from a sense of duty as a watchman on the city walls. And I publish the result simply as a sentinel would sound an alarm if he saw the enemy peeping out of an ambuscade. I am thankful that the warning has been faithfully sounded from Leeds, and I cannot but re-echo it from Bath. We may be condemned by some Christian people as only exciting a curiosity which it would be desirable to suppress. But in answer to this, I simply refer to the almost universal extent to which table-turning has been already carried. We cannot suppress the curiosity, but we may warn them against the danger of indulging in it. If a parent saw his children playing over the hole of the asp, would he leave them to their amusement, from the fear of exciting still greater curiosity by interference? I judge not. Neither could a Christian minister, who deserves the name, discover that his people were unconsciously playing with evil spirits, and fail to give them warning."

We conceive that no Christian minister who thus abuses his sacred functions is fit to be the spiritual guide and preceptor of any congregation. His facts and his reasoning are alike abhorrent to common sense.

Of course the simple experiments of Faraday amount to nothing in the eyes of this table-moving fanatic; so he has the impudence to declare—

"Professor Faraday never could have witnessed anything of the kind, or he could not for a moment entertain his physical theory as an explanation. He speaks of unconscious pressure being the cause. I say, let any number of persons press as they please, not only unconsciously, but consciously, voluntarily and openly, and they could produce nothing of the kind. Moreover, the most violent movements were often produced without the slightest pressure."

We will not pollute our pages with further extracts. The name of God Almighty is desecrated in every page, in order that these ludicrous blasphemies should be masqueraded in the garb of religion! We sincerely trust that the good people of Bath will not allow their Church to be thus desecrated. Better the Rev. E. Gillson were dressed up as harlequin than that he should wear a surplice and a gown for the dissemination of such heresies. Better it were for him to visit wakes and country fairs, than profane the Church with his antics. There is not a carpenter in any village that could not put the reverend gentleman in the way of ridding himself of such delusions; but ridicule is insufficient —a heavier punishment should be administered by the Ecclesiastical Court! Where is the Bishop of the Diocese! We sincerely hope this pamphlet will find its way into the hands of his lordship! *Verb. Sat.*

---

